# Morphological and molecular characters of *Scutellonema brachyurus* (Steiner, 1938) Andrássy, 1958 from South Africa

**DOI:** 10.21307/jofnem-2021-027

**Published:** 2021-03-11

**Authors:** Ebrahim Shokoohi

**Affiliations:** University of Limpopo, Green Biotechnologies Research Centre of Excellence, Private Bag X1106, Sovenga 0727, South Africa

**Keywords:** LSU rDNA, Morphology, mtDNA, *Scutellonema*, South Africa, Phylogeny

## Abstract

During a survey on plant-parasitic nematodes from South Africa, *Scutellonema brachyurus* was recovered from soil samples collected around the rhizosphere of wild grass in the North West and Limpopo provinces. This species characterized by a hemispherical lip region with four to six annuli, basal lip’s annuli with longitudinal incisures, body length 696–904 µm (a = 25.1–33.5; b = 5.0–7.2; c = 48.9–75.3; c’ = 0.5–0.9; V = 55–60), stylet 21–27 µm length, tail rounded with 10–19 µm length and spermatheca nonfunctional and male absent. The nblast analysis based on the D2-D3 segment of 28 S rDNA placed South African populations of *S. brachyurus* with 98% similarity to Greece (KU059494) and 99% similarity to South African (JX472052) *S. brachyurus.* Besides, nblast of COI of mtDNA showed 98% similarity of the test species with South African populations of *S. brachyurus* (JX472096; JX472097). The phylogenetic analysis put the South African populations of *S. brachyurus* together with other *S. brachyurus* with a 100 posterior probability support. Besides, the measurements, line illustration, and scanning electron microscopy photographs are provided for *S. brachyurus* from South Africa.

The genus *Scutellonema* was established by Andrássy, 1958. This genus is a well-distributed genus across all continents (Kolombia et al., 2017; Nguyen et al., 2019; Sher, 1963; Van den Berg and Heyns, 1973). The genus *Scutellonema* with its type species *S. bradys* (Steiner and LeHew, 1933) Andrássy, 1958 was initially described in association with Yam in Nigeria. Despite many species described under *Scutellonema*, however, *S. brachyurus* (Steiner, 1938) Andrássy, 1958 was previously described from South Africa (Van den Berg and Heyns, 1973; Van den Berg et al., 2013).

The present paper reports *S. brachyurus* from natural areas of South Africa. The aims of the study were (1) to study new populations of *S. brachyurus* using morphology, and (2) to study the phylogenetic position of South African *S. brachyurus* using 28 S rDNA and mtDNA.

## Materials and methods

### Nematode extraction and processing

Rhizosphere soil samples were collected from the natural grass from North West and Limpopo provinces of South Africa. Nematode extraction was achieved using the Baermann (1917) funnel technique. Subsequent, extracted individuals were fixed with a hot 4% formaldehyde solution (except those specimens used for molecular analyses) and transferred to anhydrous glycerine utilizing the method of De Grisse (1969) and mounted on permanent glass slides.

### Light microscopy (LM)

Measurements were taken of specimens mounted on permanent slides, and De Man (1880) indices were calculated. Drawings were made using a drawing tube (camera lucida) attached to an Omax microscope (China). Pictures were taken with a Nikon Eclipse 80i light microscope provided with differential interference contrast optics (DIC) and a Nikon Digital Sight DS-U1 camera (Nikon, Tokyo, Japan). Micrographs were edited using Adobe® Photoshop® CS. The terminology used for the morphology of stoma follows the proposals by Baldwin et al. (2004).

### Scanning electron microscopy (SEM)

Specimens preserved in glycerine were selected for observation under SEM, according to Shokoohi et al. (2007). The nematodes were hydrated in distilled water, dehydrated in a graded ethanol-acetone series, critical point dried, coated with gold, and observed with a Zeiss Merlin microscope (5 kV) (Zeiss, Oberkochen, Germany).

### Statistical analysis

To evaluate the morphological variations between the nematodes isolated in this study and the *S. brachyurus* populations, principal component analyses (PCA) with different morphological traits were conducted. PCA analyses were carried out in XLSTAT (Addinsoft, 2007). Various morphometric features obtained from fixed nematodes, including an average of body length, a, b, b’, c, c’, V, stylet length, DGO, m, o, excretory pore to anterior end, neck length, mid-body diameter, anal body diameter, and tail length were included in the PCA analyses (Table 2). The morphometric measurements of the PCA analysis were taken from their original descriptions (Table 2). The measures were normalized using the XLSTAT software before its analysis (Addinsoft, 2007). The scores values were determined for each species based on each of the principal components, and the scores for the first two components were used to form a two-dimensional plot (F1 and F2) of each isolate based on the eigenvalues given by the software XLSTAT.

### DNA extraction, PCR, and phylogenetic analysis

DNA extraction was done using the Chelex method (Straube and Juen, 2013). Five specimens of each locality for *S. brachyurus* were hand-picked with a fine tip needle and transferred to a 1.5 ml Eppendorf tube containing 20 μ l double distilled water. The nematodes in the tube were crushed with the tip of a fine needle and vortexed. Thirty microliters of 5% Chelex® 50 and 2 µL of proteinase K were added to each of the microcentrifuge tubes that contained the crushed nematodes and mixed. These separate microcentrifuge tubes with the nematode lysate were incubated at 56°C for 2  and then incubated at 95°C for 10 min to deactivate the proteinase K and finally spin for 2 min at 16,000 rpm (Shokoohi et al., 2018). The supernatant was then extracted from each of the tubes and stored at –20°C. Following this step, the forward and reverse primers, D2A (5’–ACAAGTACCGTGAGGGAAAGTTG–3’), D3B (5’–TCGGAAGGAACCAGCTACTA–3’) (De Ley et al., 1999), and JB3 (5’–TTTTTTGGGCATCCTGAGGTTTAT–3’), JB4.5 (5’–TAAAGA AAGAACATAATGAAAATG–3’) (Derycke et al., 2010), were used in the PCR reactions for partial amplification of the 28 S rDNA and COI of mtDNA regions, respectively. PCR was conducted with 8μ l of the DNA template, 12.5 μ l of 2X PCR Master Mix Red (Promega, USA) for the Botswanan specimens, 1 μ l of each primer (10 pmol μ l^−1^), and ddH_2_O for a final volume of 30 μ l. The amplification was processed using an Eppendorf master cycler gradient (Eppendorf, Hamburg, Germany), with the following program: initial denaturation for 3 min at 94°C, 37 cycles of denaturation for 45 s at 94°C; 56°C and 52°C annealing temperatures for 28 S rDNA and COI, respectively; extension for 45 s to 1 min at 72°C, and finally an extension step of 6 min at 72°C followed by a temperature on hold at 4°C. After DNA amplification, 4 μ l of product from each tube was loaded on a 1% agarose gel in TBE buffer (40 mM Tris, 40 mM boric acid, and 1 mM EDTA) for evaluation of the DNA bands. The bands were stained with RedGel and visualized and photographed on a UV transilluminator. The amplicons of each gene were stored at –20°C. Finally, the PCR products were purified for sequencing by Inqaba Biotech (South Africa). Also, *Hoplolaimus galeatus* (Cobb, 1913) Thorne, 1935 (KY849910; KP230651) were selected based on Kolombia et al. (2017) for 28 S rDNA and COI of mtDNA outgroups, respectively. The ribosomal DNA sequences were analyzed and edited with BioEdit (Hall, 1999) and aligned using CLUSTAL W (Thompson et al., 1994). The length of the alignments was 1,194 bp for 28 S rDNA. Phylogenetic trees were generated using the Bayesian inference method as implemented in the program Mr. Bayes 3.1.2 (Ronquist and Huelsenbeck, 2003). The GTR + I + G model was selected using the jModeltest 2.1.10 (Darriba et al., 2012; Guindon and Gascuel, 2003). The selected model was then initiated with a random starting tree and ran with the Markov chain Monte Carlo (MCMC) for 10^6^ generations. The genetic pairwise distance was calculated using the Mega-X software (Kumar et al., 2018). The partial 28 S rDNA of *S. brachyurus* was deposited in GenBank, and their accession numbers MW504474 (Parys; North West Province), MW504475 (Rustenburg; North West Province) and MW504476 (Sovenga Hills; Limpopo Province). The partial COI of mtDNA was deposited in GenBank under accession number MW509624 (Rustenburg; North West Province).

## Result

### *Scutellonema brachyurus* (Steiner, 1938) Andrássy, 1958.

Figures 1–3

**Figure 1:**
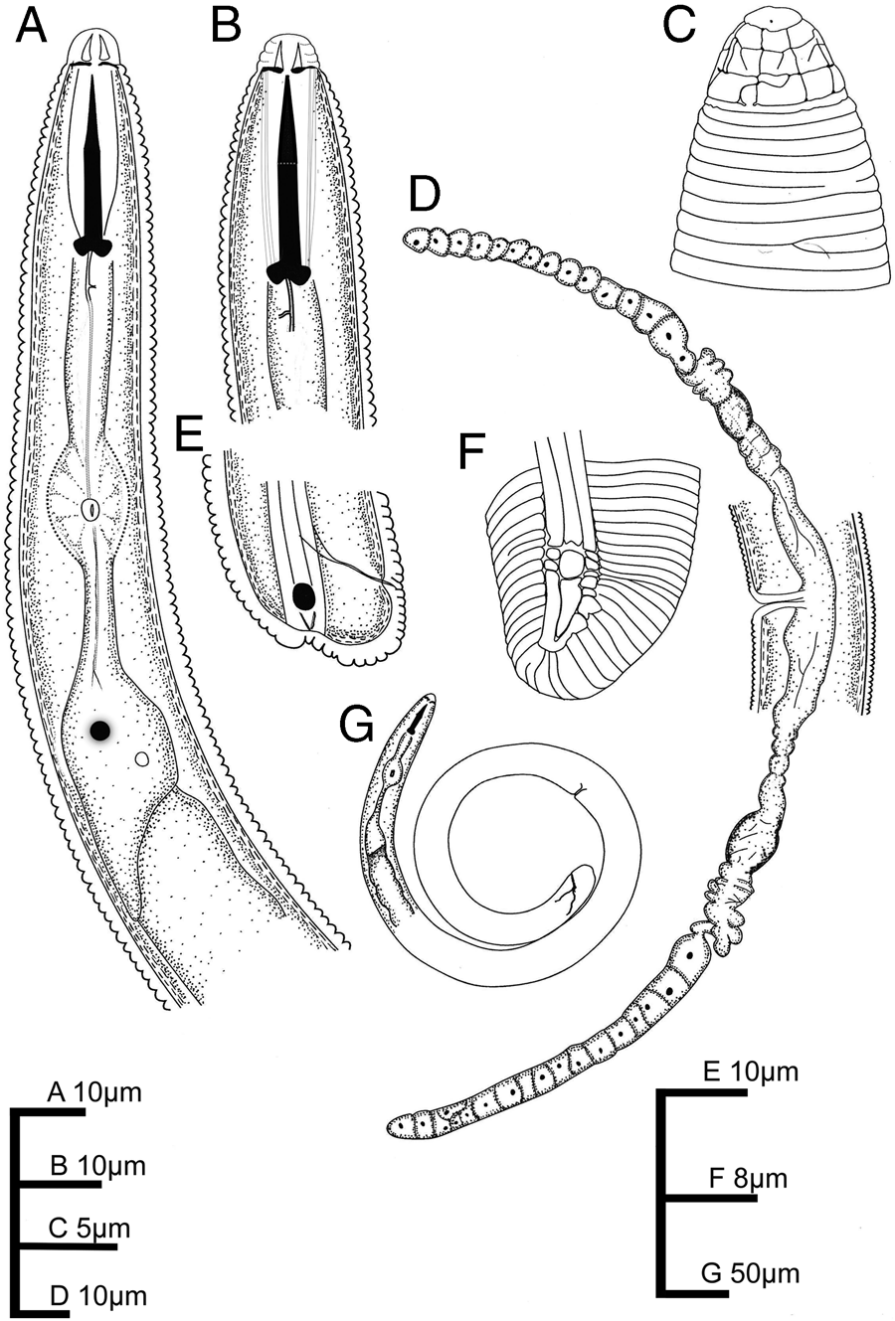
*Scutellonema brachyurus* (Steiner, 1938) Andrássy, 1958. A: Neck. B: Anterior end (stylet and dgo). C: Superficial view of lip region. D: Female reproductive system. E, F: Female posterior end. G: Entire female.

**Figure 2:**
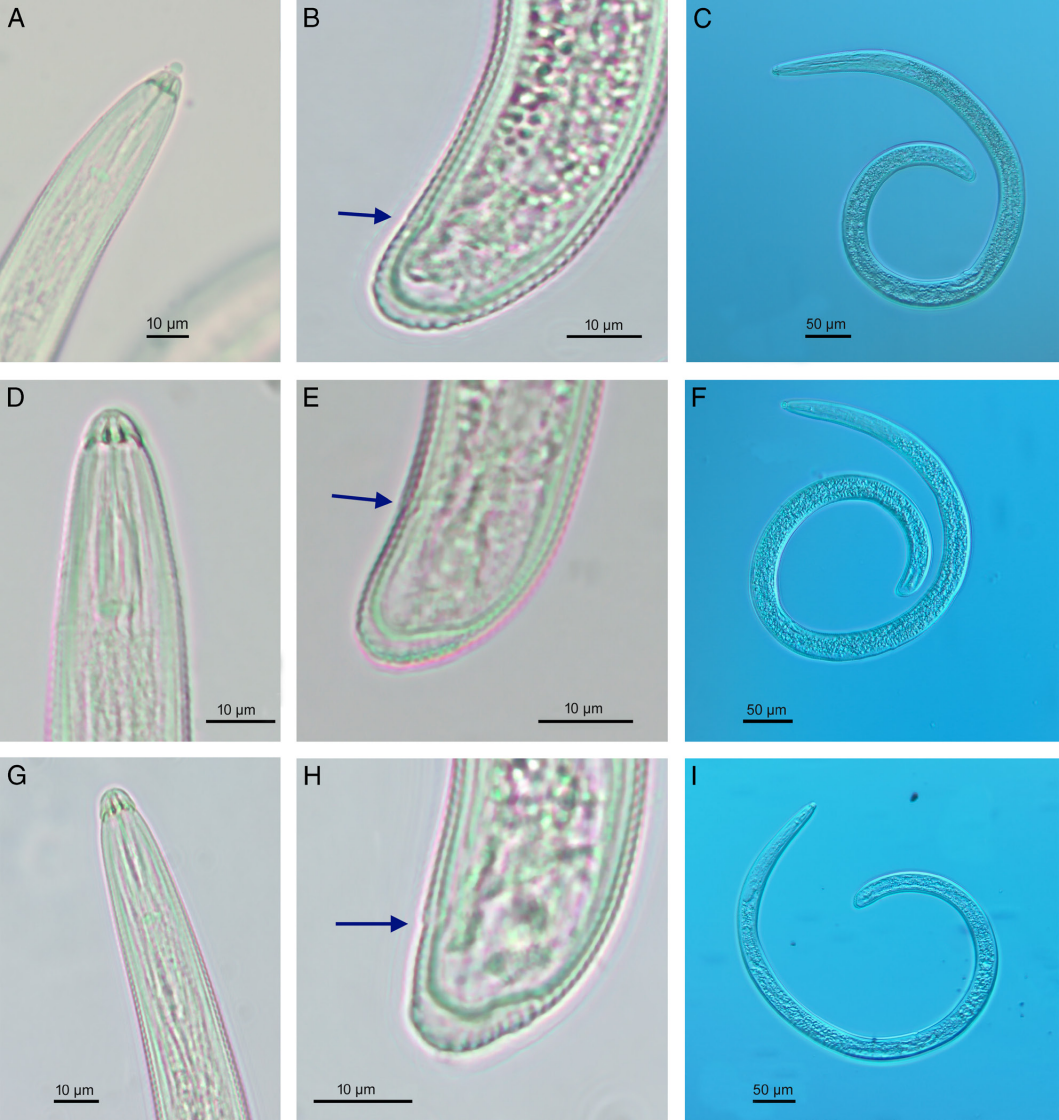
*Scutellonema brachyurus* (Steiner, 1938) Andrássy, 1958 (light microscopy). A, D, G: Lip region. B, E, H: Female posterior end (arrow indicates anus). C, F, I: Entire female. [A-C: Rustenburg population; D-F: Parys population; G-I: Sovenga Hills population].

**Figure 3:**
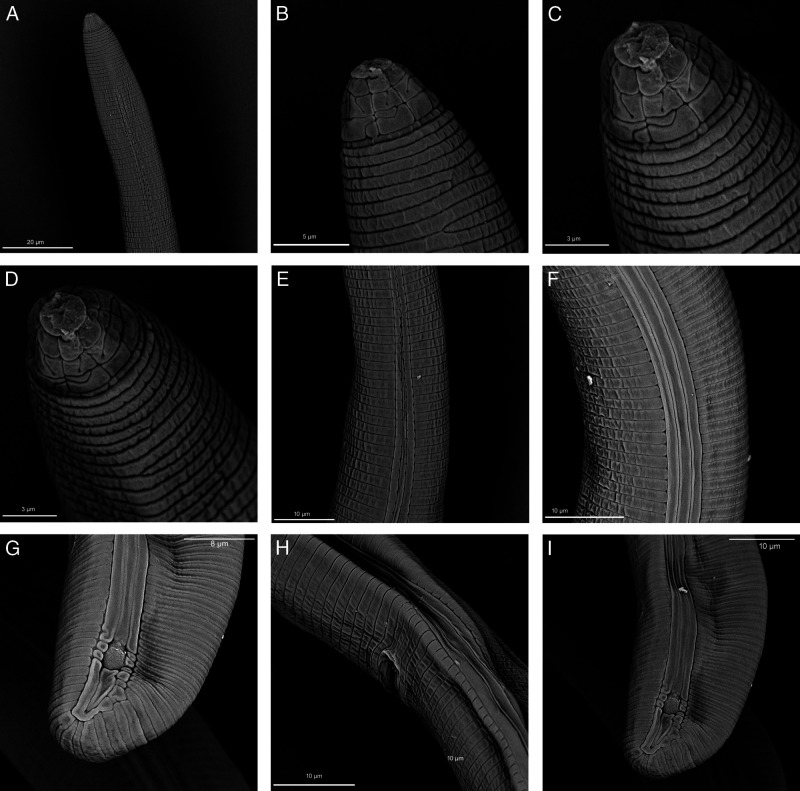
*Scutellonema brachyurus* (Steiner, 1938) Andrássy, 1958 (scanning electron microscopy). A: Neck. B-D: Lip region. E: Areolated lateral field at pharynx level. F: Normal lateral field at mid-body level. G, I: Female posterior end. H: Vaginal region.

Material examined: eight females in good condition.

Measurements: see Table 1.

**Table 1.** Measurements of *Scutellonema brachyurus* (Steiner, 1938) Andrássy, 1958 from South Africa.

Locality	Rustenburg	Parys	Sovenga Hills
n	8 ♀♀	8 ♀♀	8 ♀♀
L	736.2 ± 38.7 (696–792)	798.5 ± 16.8 (781–826)	815.6 ± 74.4 (717–904)
a	25.6 ± 0.3 (25.1–25.8)	30.8 ± 1.6 (28.5–32.3)	32.1 ± 1.6 (29.9–33.5)
b	5.4 ± 0.4 (5.0–5.8)	5.7 ± 0.1 (5.6–5.9)	6.2 ± 0.7 (5.4–7.2)
b'	6.9 ± 0.5 (6.5–7.6)	6.5 ± 0.6 (5.9–7.2)	8.0 ± 1.1 (6.8–9.5)
c	54.1 ± 4.0 (49.7–59.4)	58.9 ± 10.7 (48.9–72.5)	70.7 ± 4.2 (65.3–75.3)
c'	0.7 ± 0.0 (0.7)	0.8 ± 0.2 (0.6–0.9)	0.6 ± 0.1 (0.5–0.7)
V	57.2 ± 2.0 (55–60)	58.1 ± 0.5 (57.6–58.5)	56.8 ± 0.9 (56–58)
Lip region diameter	9.0 ± 0.9 (7–10)	8.4 ± 0.6 (7.8–9.1)	8.3 ± 0.3 (8.0–8.6)
Lip region height	5.3 ± 0.4 (4.7–5.5)	5.4 ± 0.5 (5–6)	5.6 ± 0.3 (5–6)
Conus length	9.5 ± 1.0 (9–11)	10.7 ± 0.5 (10–11)	10.3 ± 1.0 (9–11)
Shaft length	11.3 ± 1.2 (10–12)	11.6 ± 0.5 (11–12)	12.3 ± 1.0 (11–13)
Stylet knob height	2.7 ± 0.2 (2–3)	3.0 ± 0.4 (2–3)	2.7 ± 0.3 (2–3)
Stylet knob width	4.3 ± 0.7 (3.5–5.2)	5.2 ± 0.3 (4.8–5.4)	4.3 ± 1.0 (3.3–5.2)
Stylet length	23.4 ± 1.9 (21–27)	25.8 ± 1.0 (25–27)	25.5 ± 1.3 (24–27)
DGO	4.8 ± 0.5 (4–5)	4.6 ± 0.5 (4–5)	5.1 ± 0.5 (4.5–5.5)
m	41.9 ± 2.2 (39–44)	41.4 ± 1.2 (40–42)	41.1 ± 3.6 (37–44)
o	20.4 ± 2.8 (19–22)	17.8 ± 2.1 (15–20)	19.7 ± 3.3 (17–22)
Median bulb length	13.1 ± 1.9 (11–15)	12.7 ± 0.6 (12–13)	12.8 ± 2.5 (10–16)
Median bulb diameter	9.8 ± 1.0 (8.5–10)	9.2 ± 0.7 (8.7–10)	9.5 ± 1.3 (8–11)
Median bulb valve length	3.0 ± 1.0 (2–4)	2.9 ± 0.9 (2–4)	3.3 ± 0.7 (2.5–3.9)
Median bulb valve width	1.5 ± 0.3 (1.1–1.7)	1.2 ± 0.3 (1.0–1.4)	1.5 ± 0.5 (1.0–1.8)
Median bulb to anterior end	72.5 ± 0.6 (72–73)	74.3 ± 2.5 (72–77)	76.0 ± 3.6 (72–79)
Pharynx length	105.0 ± 12.1 (91–114)	108.0 ± 1.4 (107–110)	104.0 ± 2.2 (102–106)
Pharyngeal overlap	29.8 ± 2.2 (27–32)	24.0 ± 3.6 (21–28)	28.5 ± 3.9 (24–33)
Nerve ring to anterior end	89.0 ± 4.8 (86–96)	101.3 ± 8.3 (92–108)	95.3 ± 6.8 (90–105)
Excretory pore to anterior end	113.6 ± 5.1 (106–116)	118.5 ± 1.7 (117–121)	111.0 ± 6.2 (104–116)
Neck length (stoma + pharynx)	137.5 ± 9.4 (121–148)	140.3 ± 2.6 (138–144)	135.2 ± 7.1 (125–144)
Neck base diameter	24.0 ± 2.6 (22–27)	22.8 ± 1.7 (21–25)	23.7 ± 1.5 (22–25)
Mid-body diameter	28.3 ± 1.0 (27–29)	25.8 ± 1.5 (25–28)	25.8 ± 1.3 (24–26)
Anal body diameter	19.1 ± 0.9 (17–21)	17.2 ± 0.8 (16–18)	18.4 ± 1.5 (16–20)
Annulus width	1.4 ± 0.2 (1.3–1.6)	1.4 ± 0.2 (1.2–1.7)	1.3 ± 0.1 (1.2–1.4)
Lateral field width	4.9 ± 0.5 (4.5–5.6)	4.9 ± 1.3 (4.0–5.8)	4.5 ± 0.7 (3.8–5.2)
Vagina length	11.5 ± 1.0 (11–13)	12.0 ± 0.8 (11–13)	11.0 ± 0.8 (10–12)
Anterior genital branch length	115.9 ± 16.4 (104–128)	–	156.0 (n = 1)
Posterior genital branch length	139.8 ± 7.4 (135–145)	–	–
Spermatheca length	11, 12 (n = 2)	–	–
Spermatheca width	9.0, 10 (n = 2)	–	–
Scutellum width	3.9 ± 0.6 (3.2–4.4)	2.8 ± 1.0 (2.1–4.3)	3.4 ± 0.9 (2.6–4.4)
hyaline length	3.5 ± 0.7 (2.8–4.6)	2.9 ± 0.6 (2.0–3.4)	2.8 ± 0.7 (2.2–3.6)
Tail length	12.8 ± 1.4 (10.5–14.0)	14.4 ± 2.4 (11–17)	13.2 ± 3.4 (10–19)

All measurements are in μ m and in the form: mean ± s.d. (range).

**Table 2.** Loading factor of the variables of different populations of *Scutellonema brachyurus* (Steiner, 1938) Andrássy, 1958.

	F1	F2
L	−0.847	−0.076
a	0.054	−0.703
b	−0.043	−0.472
b'	−0.730	0.284
c	0.735	−0.008
c'	−0.192	−0.842
V	0.957	0.196
m	0.696	0.024
o	0.913	−0.120
Stylet L	−0.371	0.476
DGO	0.816	0.119
Excretory pore to ant. end	0.272	−0.247
Neck length	−0.433	0.383
Maximum body diameter	0.116	0.931
Anal body diameter	−0.640	0.396
Tail length	−0.690	−0.535

### Description

#### Female

Body spiral after heat relaxation. Lip region slightly offset with body contour, with bearing three (Figure 3) to five annuli. The basal annuli with *ca* eight blocks (four visible in ventral view; Figure 3). Amphids oval shaped on the small block of the third lip region annuli, located on the lip region’s lateral side. Lateral fields with four incisures, the three bands are 4.9 ± 0.5 (4.5–5.6) µm wide at mid-body, approximately 16–21% of the corresponding bod diameter. Regular areolation of lateral fields observed in the pharyngeal region and scutellum. Cuticle 1.0–1.5 µm thick, clearly annulated, one annulus 1.4 ± 0.2 (1.3–1.6) µm wide at mid-body. Body without longitudinal striations in any region. Stoma comprises cheilostom ( = conus) 39–44% of the stoma length, gymnostom ( = almost part of the shaft) 48–51% of the stoma length, and prostegostom ( = posterior part of the shaft and knobs) 17–21% of the stoma length. Stylet moderately robust, 2–3 times longer than labial region diameter. Basal knobs rounded slightly forward-directed, 4.3 ± 0.7 (3.5–5.2) µm wide. Anterior and posterior cephalids not seen. Dorsal pharyngeal gland opening 4.8 ± 0.5 (4.0–5.0) µm posterior to stylet base. Pharynx tylenchoid with procorpus cylindrical, with slight depression just anterior to the median bulb. Median pharyngeal bulb well developed, oval, 8.5–10 × 11–15 µm, valvular apparatus 2.0–4.0 m long, located at 54.5 ± 4.3 (52–60) % of neck length. Isthmus shorter than corpus, encircled by nerve ring at mid-point. Pharyngeal glands short, with three nuclei, slightly overlapping intestine dorsally. Nerve ring enveloping isthmus at the middle, at 61–73% of the neck from anterior end. Excretory pore usually located at posterior part of isthmus, pharyngeal bulb or near pharyngo-intestinal junction level, at 77–96% of the neck length from anterior end. Hemizonid distinct, 2–3 annuli long, located 2–5 annuli anterior to excretory pore. The reproductive system with both genital branches well-developed; anterior branch 115.9 ± 16.4 (104–128) µm long, posterior branch 139.8 ± 7.4 (135–145) µm long. Vulva posterior to mid-body, with epiptygma, folded into vagina. Vagina with internal walls slightly sclerotized, 11.5 ± 1.0 (11–13) µm long. Spermatheca slightly oval, 12 × 9 µm (*n*  = 1), without sperm, ovaries with a single row of oocytes. Intestine not overlapping the rectum. Rectum 13–15 µm long. Scutellum moderate, 3.9 ± 0.6 (3.2–4.4) µm, two to three annuli anterior or three to six annuli posterior to the anus. Tail short, rounded, 12.8 ± 1.4 (10.5–14.0) µm long, with 7–14 annuli at ventral side. Hyaline at tail tip 3.5 ± 0.7 (2.8–4.6) µm wide.

#### Male

Not found.

### Locality and habitat

Specimens of *S. brachyurus* were collected in North West Province (Rustenburg: S 25° 40’ 55.215”, E 27° 14’ 36.028” and Parys: S 26° 54’ 37.336”, E 27° 27’ 24.582”), and Limpopo Province (Sovenga Hill; S 23° 53’ 41.763”, E 29° 44’ 21.292”), around the rhizosphere of natural grass.

#### Remarks

Morphologically, there are no significant differences between the examined specimens of *S. brachyurus* with the original description (Andrássy, 1958; Steiner, 1938) and those studied by Sher (1963). However, compared with the South African specimens studied by Van den Berg and Heyns (1973), they differ in body length (0.6–0.9 vs 0.5–1.0 mm). In comparison with South African material examined by Van den Berg et al. (2013), they differ in stylet length (21–27 vs 25.5–32 µm). However, compared with the American populations of *S. brachyurus* (Van den Berg et al., 2013), they differ in body length (696–904 vs 611–805 µm), stylet length (vs 27–30.5 µm), and tail length (10–19 vs 7.5–14 µm). Compared with specimens studied by Tzortzakakis et al. (2016), they differ in body length (vs 640–760 µm). Compared with the population studied by Nguyen et al. (2019), they differ in tail length (vs 9.8–13.3 µm) and block number of the basal lip’s annuli (8–12 vs 8–20).

### PCA analysis

**Figure 4:**
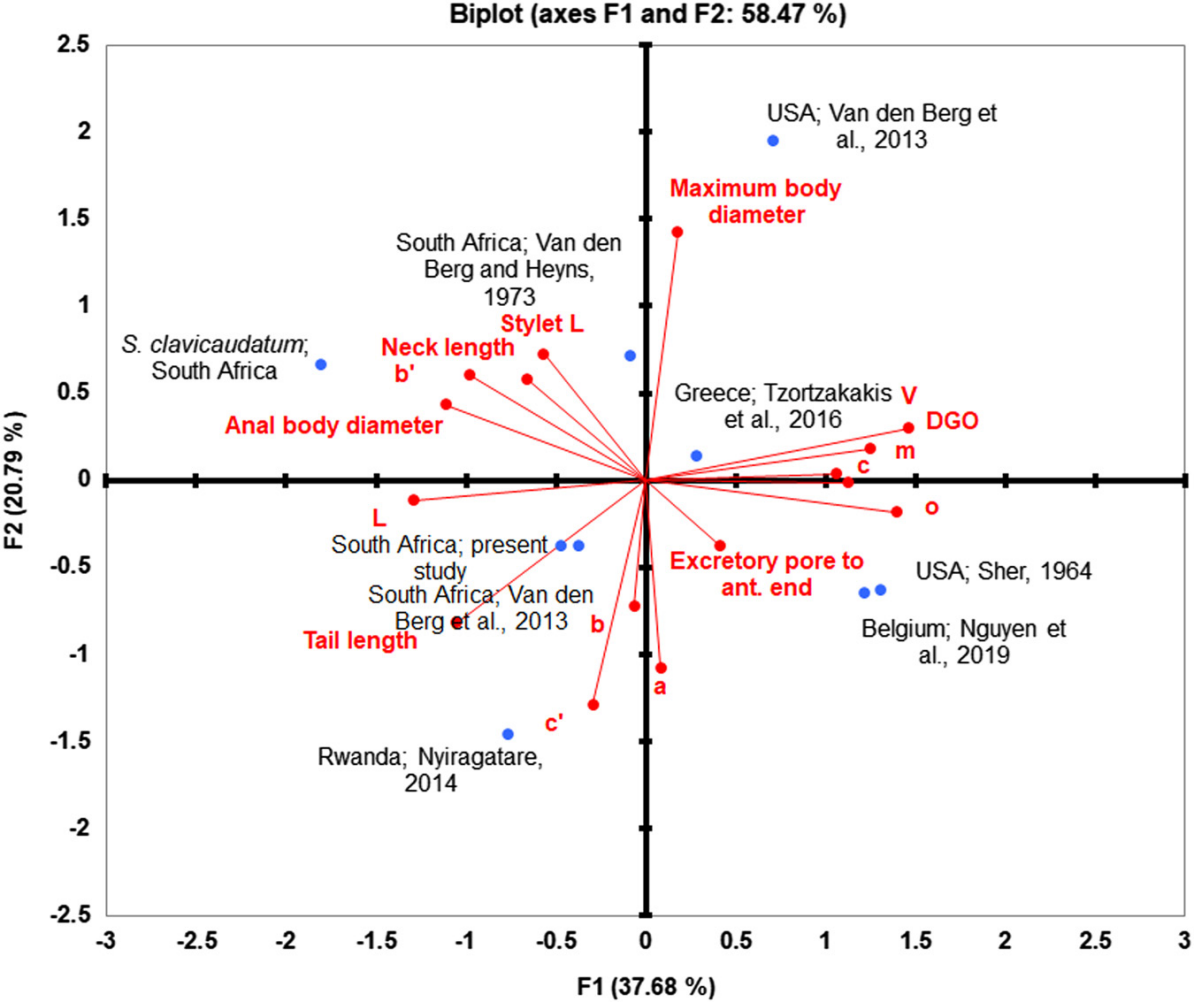
PCA analysis of *S. brachyurus* populations.

Principal component analysis using morphometric features of the females of *S. brachyurus*, including the populations from South Africa, showed that *S. brachyurus* has morphometric variation. Specifically, we have selected 16 morphometric variables for the mature females, which allowed for clear differentiation of different populations for *S. brachyurus* (Figure 4).

An accumulated variability of 58.47% was observed in female-based PCA, specifically, 37.68% in the F1 and 20.79% in the F2. Almost all the variables showed a positive correlation between them and were responsible for the significant variability of the F1, while some variables such as L, b, b’, c’, stylet length, neck length, anal body diameter and tail length displayed a negative correlation (Figure 4, Table 2). The variables V (*r* = 0.957), o (*r* = 0.913), and DGO (*r* = 0.816) were responsible for the significant variability of the F1 (Figure 4). Regarding the F2, maximum body diameter (*r* = 0.931) had the most contribution to the variability and showed a positive and high correlation (Table 2). Overall, the result indicated that the population from South Africa stands together. In contrast, the populations from America and Belgium grouped together (Table 3 and Figure 4). The result indicated low variability within the South African population for *S. brachyurus*. The result indicated that *S. clavicaudatum* Van den Berg et al., 2017 place separately than other *S. brcahyurus* populations.

**Table 3.** Factor score for different populations of *Scutellonema brachyurus* (Steiner, 1938) Andrássy, 1958 and *S. clavicaudatum*.

Observation	F1	F2
*S. brachyurus*; South Africa; present study	−1.167	−0.680
*S. brachyurus*; Rwanda; Nyiragatare, 2014	−1.890	−2.663
*S. brachyurus*; USA; Sher, 1964	2.978	−1.170
*S. brachyurus*; South Africa; Van den Berg and Heyns, 1973	−0.209	1.312
*S. brachyurus*; Belgium; Nguyen et al., 2019	3.209	−1.150
*S. brachyurus*; Greece; Tzortzakakis et al., 2016	0.692	0.265
*S. brachyurus*; South Africa; Van den Berg et al., 2013	−0.920	−0.684
*S. brachyurus*; USA; Van den Berg et al., 2013	1.747	3.554
*S. clavicaudatum*; South Africa; Van den Berg et al., 2017	−4.440	1.216

#### DNA characterization

The genes 28 S rDNA and COI of mtDNA for *S. brachyurus* yielded 710–714 and 401 bp, respectively. Nblast of the 28 S rDNA showed 99% identity with the South African population (acc. nrs: JX472050; JX472051; JX472052) of *S. brachyurus.* Compare with a population of *S. truncatum* (acc. nr: KX959272) showed a 90% similarity. Regarding COI of mtDNA, Nblast showed 98% similarity with South African populations of *S. brachyurus* (acc. nrs: JX472096; JX472097; JX472098). Compared with *S. truncatum* from Benin (MG973138), our sequence showed 79% similarity, whereas another population of *S. truncatum* (KX959307) from Botswana showed 82% similarity with tested *S. brachyurus* from South Africa. Genetic pairwise distance (Table 4) for 28 S rDNA marker, indicated the lowest (0.01) and the highest range (0.07) among *S. brachyurus*. However, populations belong to type A (American populations; one South African population) having 0.01–0.03, whereas the genetic distances among the type B (South African populations) ranges from 0.06–0.07. In addition, *S. clavicaudatum* showed the same genetic distance estimation with type B of *S. brachyurus*.

**Table 4.** Genetic pairwise distance estimating for type A and B of different populations of *S. brachyurus* (numbers 1–12) and *S. clavicaudatum* (numbers 13–17).

Seq number	Accession number	Country	type	1	2	3	4	5	6	7	8	9	10	11	12	13	14	15	16	17
1	MW504475	South Africa	A		0.01	0.01	0.01	0.01	0.01	0.01	0.01	0.01	0.01	0.01	0.01	0.01	0.01	0.01	0.01	0.01
2	KX959260	USA	A	0.02		0.00	0.01	0.00	0.00	0.00	0.01	0.01	0.01	0.01	0.01	0.01	0.01	0.01	0.01	0.01
3	KU059494	USA	A	0.02	0.01		0.01	0.00	0.00	0.00	0.01	0.01	0.01	0.01	0.01	0.01	0.01	0.01	0.01	0.01
4	JX472047	USA	A	0.03	0.02	0.02		0.01	0.01	0.01	0.01	0.01	0.01	0.01	0.01	0.01	0.01	0.01	0.01	0.01
5	DQ328753	USA	A	0.01	0.01	0.01	0.02		0.00	0.00	0.01	0.01	0.01	0.01	0.01	0.01	0.01	0.01	0.01	0.01
6	EU280787	USA	A	0.02	0.01	0.01	0.02	0.00		0.00	0.01	0.01	0.01	0.01	0.01	0.01	0.01	0.01	0.01	0.01
7	FJ485645	USA	A	0.02	0.01	0.01	0.02	0.00	0.01		0.01	0.01	0.01	0.01	0.01	0.01	0.01	0.01	0.01	0.01
8	MW504474	South Africa	B	0.06	0.07	0.08	0.08	0.07	0.07	0.08		0.00	0.01	0.01	0.01	0.01	0.01	0.01	0.01	0.01
9	MW504476	South Africa	B	0.07	0.07	0.07	0.07	0.08	0.08	0.08	0.01		0.00	0.00	0.00	0.01	0.01	0.01	0.01	0.01
10	JX472057	South Africa	B	0.07	0.07	0.06	0.06	0.07	0.07	0.08	0.02	0.01		0.00	0.00	0.01	0.01	0.01	0.01	0.01
11	JX472056	South Africa	B	0.07	0.07	0.06	0.06	0.07	0.07	0.08	0.02	0.01	0.00		0.00	0.01	0.01	0.01	0.01	0.01
12	JX472048	South Africa	B	0.07	0.07	0.06	0.06	0.07	0.07	0.08	0.02	0.01	0.01	0.01		0.01	0.01	0.01	0.01	0.01
13	KX959268	South Africa	–	0.08	0.08	0.08	0.07	0.08	0.08	0.09	0.08	0.07	0.06	0.06	0.06		0.00	0.01	0.01	0.01
14	KX959267	South Africa	–	0.07	0.08	0.07	0.07	0.08	0.08	0.08	0.07	0.06	0.06	0.06	0.05	0.01		0.01	0.01	0.01
15	KX959269	South Africa	–	0.07	0.08	0.08	0.07	0.08	0.08	0.09	0.07	0.06	0.06	0.06	0.06	0.02	0.02		0.01	0.01
16	KX959266	South Africa	–	0.07	0.08	0.08	0.07	0.08	0.08	0.09	0.06	0.05	0.05	0.05	0.04	0.05	0.05	0.04		0.00
17	KX959265	South Africa	–	0.07	0.08	0.08	0.07	0.08	0.08	0.09	0.06	0.05	0.05	0.05	0.04	0.05	0.05	0.04	0.01	

Black colour genetic pairwise distance; blue colour standard error).

#### Phylogenetic analysis

**Figure 5:**
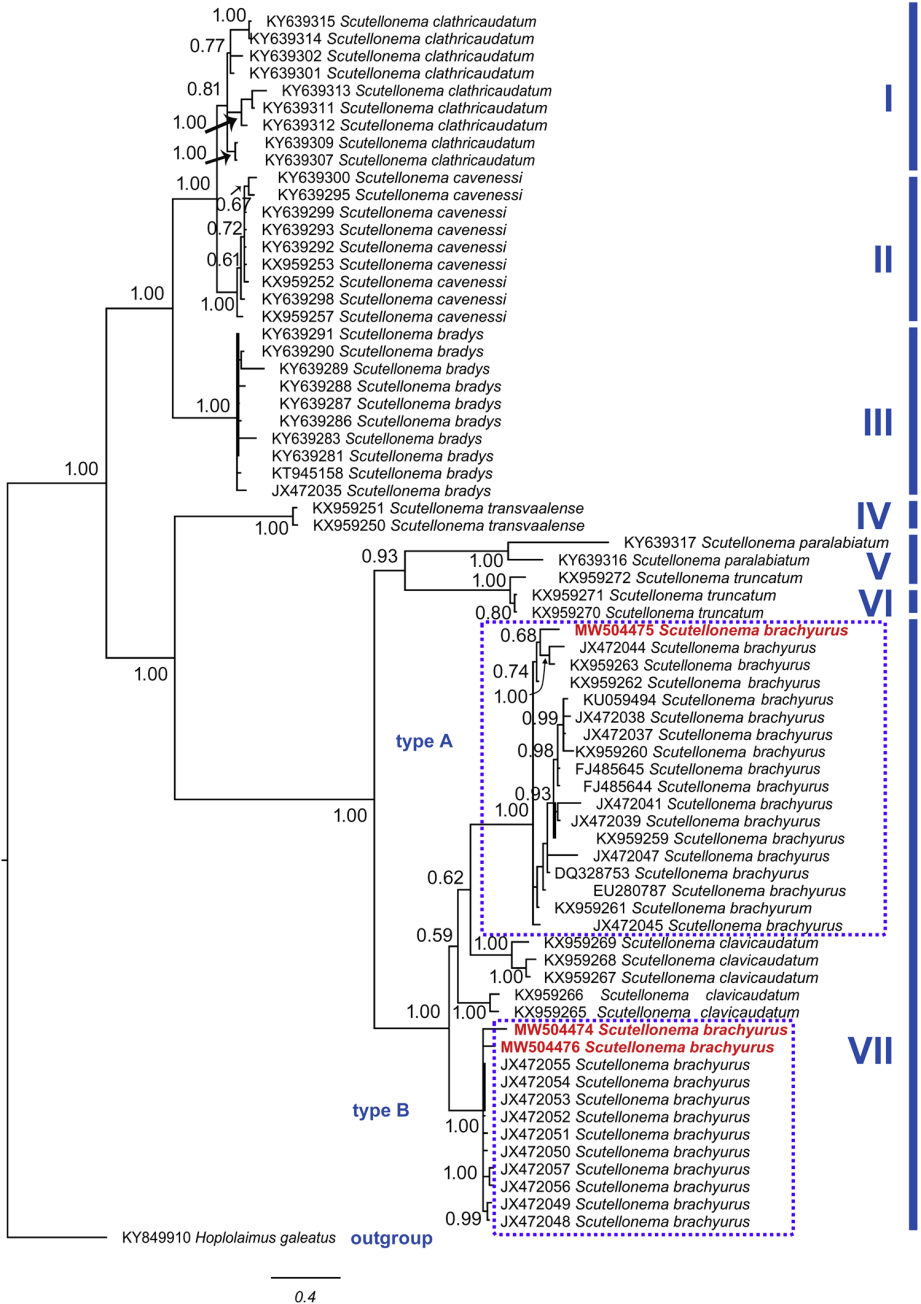
The Bayesian tree inferred from known and newly sequenced *S. brachyurus* from South Africa based on 28 S rDNA region.

The Bayesian inference tree of 28 S rDNA of *Scutellonema* species (Figure 5) grouped them into seven clades, including (I) *S. clathricaudatum* Whitehead, 1959 with 1.00 posterior probability; (II) *S. cavenessi* Sher, 1964 with 1.00 posterior probability; (III) *S. bradys* with 1.00 posterior probability; (IV) *S. transvaalense* Van den Berg, 1981 with 1.00 posterior probability; (V) *S. paralabiatum* Siddiqi and Sharma, 1994 with 1.00 posterior probability; (VI) *S. truncatum* Sher, 1964 with 1.00 posterior probability; and (VII) *S. clavicaudatum* and *S. brachyurus* with 1.00 posterior probability.

**Figure 6:**
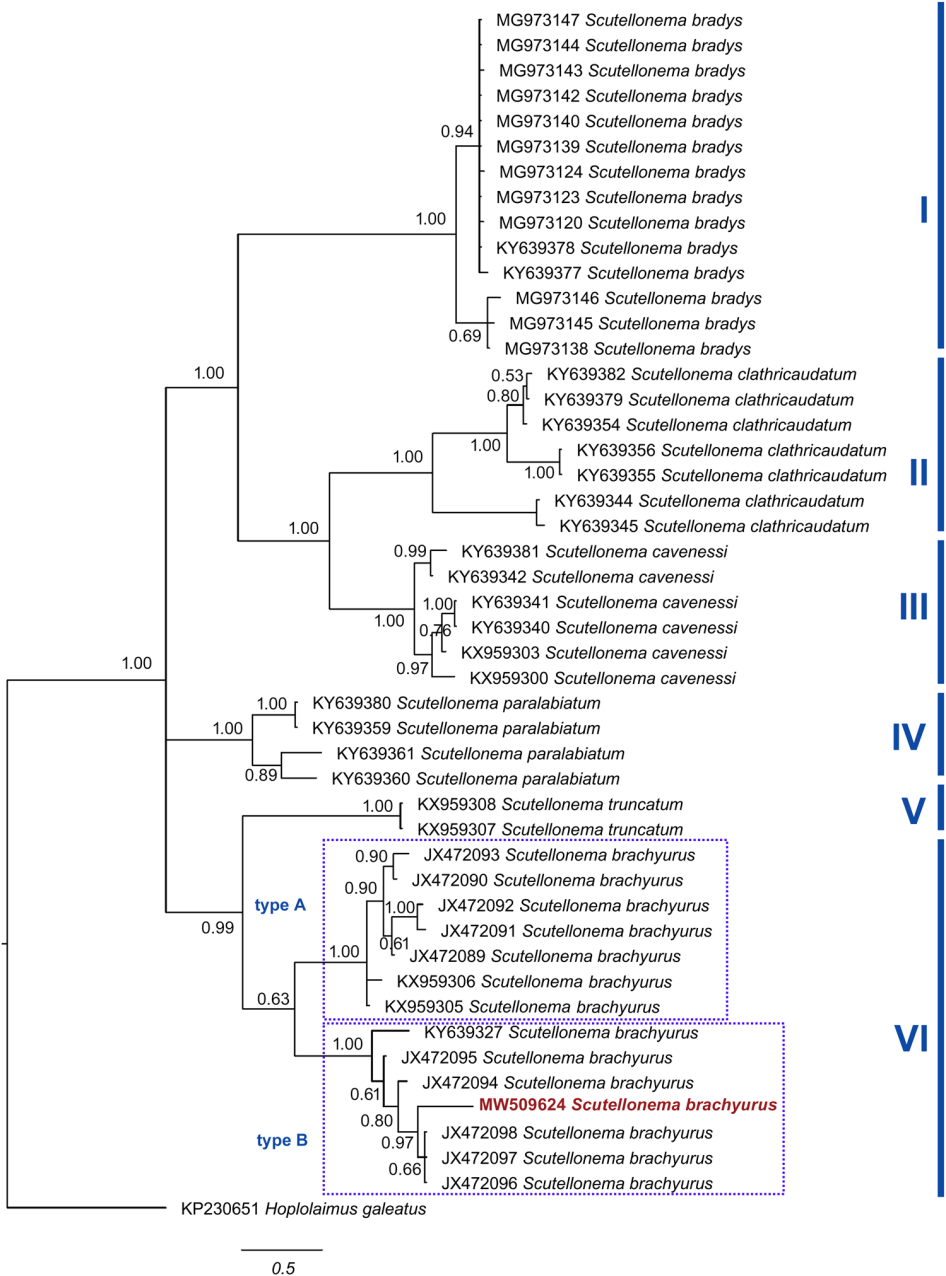
The Bayesian tree inferred from known and newly sequenced *S. brachyurus* from South Africa based on COI of mtDNA region.

The Bayesian inference tree of COI of mtDNA of *Scutellonema* species (Figure 6) grouped them into six clades, including (I) *S. bradys* with 1.00 posterior probability; (II) *S. clathricaudatum* with 1.00 posterior probability; (III) *S. cavenessi* with 1.00 posterior probability; (IV) *S. paralabiatum* with 1.00 posterior probability; (V) *S. truncatum* with 1.00 posterior probability; (VI) *S. transvaalense* with 1.00 posterior probability; and (VII) *S. brachyurus* with 0.63 posterior probability.

## Discussion

Our phylogenetic analysis using 28 S rDNA and COI of mtDNA (Figures 4 and 5) placed the South African *S. brachyurus* populations, together with the other *S. brachyurus*. Additionally, based on 28 S rDNA phylogeny, *S. brachyurus* and *S. clavicaudatum* place together in a clade with support of a 1.00 posterior probability value. However, they clearly distinguish in lip region (lips annuli present in *S. brachyurus* vs lips annuli absent in *S. clavicaudatum*) and tail (cylindrical in *S. brachyurus vs* clavate in *S. clavicaudatum*). The lip region is differentiated into small blocks in *S. brachyurus*, whereas six large rectangular blocks are present in *S. clavicaudatum* (see Van den Berg et al., 2017). The PCA result indicated that *S. clavicaudatum* places separately from *S. brachyurus*. The genetic distance revealed South African *S. brachyurus* (type B) and *S. clavicaudatus* share the same range (0.07–0.08). Based on ITS and 28 S rDNA phylogeny, these two species are placed within a clade (Van den Berg et al., 2017). Albeit, the COI of mtDNA is not available yet for *S. calvicaudatum*. Therefore, a more molecular marker is needed to resolve the relationship between the two discussed species.

The previous studies revealed two types of *S. brachyurus* (Kolombia et al., 2017; Van den Berg et al., 2013, 2017), including type A (USA) and type B (South Africa). However, they differ in tail length (7.5–14 in the American population vs 7.5–23 in the South Africa population; Van den Berg et al., 2013). In addition, the American basal lip’s annuli for *S. brachyurus* divided into more expanded blocks than the South Africa population (Van den Berg et al., 2013). The same characters were observed in the Rustenburg population’s present study (SEM photograph), which confirm they belong to type B.

The PCA results (Van den Berg et al., 2013) also showed that American and South African populations for *S. brachyurus* stand separately. This is in agreement with the obtained result of the present study. However, the phylogenetic result indicated that one of the South African sequences stand together with type A. This is in contradiction with the result obtained by Van den Berg et al. (2013). Despite the morphological differences of the South African and American populations of *S. brachyurus*, the present study’s result indicates that dividing the *S. brachyurus* into type A and B needs to be reconsidered. A population of *S. brachyurus* from South Africa is placed in the same group as the American population. Besides, the PCA results also revealed that American populations of *S. brachyurus* also having a variation. Additionally, genetic pairwise distance revealed type A (0.01–0.03) and B (0.06–0.07) are different. Overall, the molecular study using 28 S rDNA and COI of mtDNA indicated that different populations of *S. brachyurus* place under the same clade. However, populations that belong to this species make a cryptic species need to study the populations deeply. The 28 S rDNA and COI of mtDNA markers demonstrated an admirable option for diagnosing species belong to *Scutellonema*. However, type A and B phylogenetic position remains unresolved, as indicated by Van den Berg et al. (2017). Four permanent microscope slides of *S. brachyurus* were deposited in the Nematology laboratory collection of the University of Limpopo, South Africa. In conclusion, the ecological behavior of *S. brachyurus* in terms of the economic impact on the crops needs to be investigated.
